# Training Resources Targeting Social Media Skills to Inform Rehabilitation for People Who Have an Acquired Brain Injury: Scoping Review

**DOI:** 10.2196/35595

**Published:** 2022-04-28

**Authors:** Melissa Brunner, Rachael Rietdijk, Leanne Togher

**Affiliations:** 1 Faculty of Medicine and Health University of Sydney Eora Nation, Camperdown Australia

**Keywords:** brain injury, social media, training, social communication, scoping review

## Abstract

**Background:**

In 2020 and 2021, people increasingly used the internet to connect socially and professionally. However, people with an acquired brain injury (ABI) experience challenges in using social media, and rehabilitation professionals have reported feeling underprepared to support them in its use. To date, no review of social media skills training to inform ABI rehabilitation has been conducted.

**Objective:**

This scoping review aimed to examine research on interventions addressing social media skills and safety, with a focus on people living with health conditions; free web-based resources for the general public on social media skills training; and currently available online support groups for people with ABI.

**Methods:**

An integrative scoping review was conducted, with a systematic search strategy applied in March and November 2020 across OvidSP (MEDLINE, AMED, PsycINFO, and Embase), Scopus, Web of Science, CINAHL, Google Scholar, Google, and Facebook. The data collected were critically appraised and synthesized to describe the key content and features of social media training resources.

**Results:**

This review identified 47 peer-reviewed academic articles, 48 social media training websites, and 120 online support groups for people with ABI. A key recommendation was interactive training with practical components addressing cybersafety, how to use platforms, and how to connect with others. However, no social media training resources that were relevant and accessible for people with ABI were identified.

**Conclusions:**

Training resources to support people with ABI in safely using social media are limited. The key content to be addressed and the features to be incorporated into web-based social media training were determined, including the need for interactive training that is co-designed and safe and incorporates practical components that support people with ABI. These findings can be used to inform the development of web-based evidence-based support for people with ABI who may be vulnerable when participating in social media.

## Introduction

### Background

Connecting with others and having conversations is an integral part of being a person in our society, where being social through developing and maintaining relationships is customary for many [1]. From birth, we learn through exploration [2]; as we learn, our brains discover how to adapt and accomplish more complex tasks [3]. Being social and having conversations are complex tasks that demonstrate the ability of our brain to learn and develop over time through our interactions with one another [4]. Throughout 2020 and 2021, because of restrictions being placed on real-life interactions physically, we observed immense changes in the way we interacted globally, with people increasingly using the internet to connect socially and professionally [5]. Using social media allows people living in this digital age to connect with others locally and internationally in various ways [6,7]. In addition to using social media to communicate with peers [6], communities have increasingly been strategically connecting on social media for climate change activism [8], disaster response and recovery [9], and global health issues relating to the COVID-19 pandemic [10]. These web-based communities include those who have communication difficulties resulting from brain injury.

An acquired brain injury (ABI) is defined as an injury to the brain that occurs after birth and is not related to congenital disorders, developmental disabilities, or degenerative processes [11] and can be the result of both traumatic and nontraumatic causes (eg, through a stroke, tumor, infection, or trauma). In 2016, there was a global incidence of 276 million people who were living with an ABI [12]. More recent epidemiological studies indicate that this number is increasing substantially, with reports of 12 million people who have a stroke [13] and 69 million people who sustain a traumatic brain injury (TBI) [14] annually. For people who experience an ABI, changes in their cognitive function alter their executive functioning and social communication skills [15]. As a result, many individuals have difficulties living independently, returning to work or study, and navigating their interpersonal relationships [16-19]. For those who experience aphasia (ie, “a communication disability due to an acquired impairment of language modalities caused by focal brain damage”) [20] or significant physical disability whereby speech clarity is difficult to achieve (ie, dysarthria or dyspraxia) [21], changes in communication after their injury can be marked, with social communication difficulties immediately apparent to their communication partners. However, for people who experience cognitive-communication disorders (commonly occurring after a right hemisphere stroke or TBI) [22], changes in a person’s social communication can present with more subtlety. For example, an individual with cognitive-communication difficulties may be able to have a conversation, yet they may either be verbose or alternatively have impoverished conversational skills (ie, they talk too much or too little in a conversation) [23]. Although subtle, these changes in their interactions and conversations resulting from cognitive-communication difficulties can have a dramatic impact on their life [24]. Within a year of their brain injury, many people lose their friends, and their remaining relationships can be strained [25]. This means that they are often socially isolated and can feel disconnected [26] and have difficulty engaging with their wider social networks as well as in their close relationships [27].

Using social media may present an opportunity to reduce social isolation after an ABI, offering an important way for people to connect with family, friends, and the broader community [28]. Social media platforms can provide a view of the lives of others, opportunities to explore topics of personal interest, and an avenue for people to interact with other users that may not be possible or accessible in their real-world environments. The use of social media may allow people to prepare for their communicative interactions without the cognitive overload that face-to-face interactions may present [29]. Asynchronous communications in social media may reduce pressure regarding the need for immediate responses and eliminate the need to use and interpret social nonverbal skills in real time [29]. In addition, there is the ready accommodation of spelling and grammatical errors observed in social media posts among the general public [30] as well as greater awareness of the need to reduce linguistic bigotry [31], as seen in fierce criticism of social media examples such as the Instagram account @celeb_spellcheck that mocks spelling and grammatical errors of other users [32]. Using social media can also give people the opportunity to interact with others on the web without disclosing their history of brain injury, or alternatively, it can provide a platform for individuals to advocate for themselves (and the ABI community) as people living with an ABI [29].

Although social media offers many opportunities for connection, it also presents safety risks to individuals [33], with >50% of the general public having experienced or observed negative social media interactions [34]. People who have physical impairments, intellectual disabilities, and specific chronic diseases are often specifically targeted on the web, which can result in depression, anxiety, and distress [35]. Cyberharassment incidents against people who have a disability occur with greater frequency than for the general public, with some cyberscammers pretending to have a disability themselves to insinuate themselves into the lives of their potential targets [35,36]. Although people with a TBI use social media for connection and communication, few report having received formal support during rehabilitation in social media skills or safety, and those who had been cyberscammed had not been offered training after these distressing experiences [37]. Limitations in our individual abilities to use social media, communication difficulties and patterns, and the social networks we interact with are sources of vulnerability in web-based interactions [38]. In seeking connection on the web, the vulnerability of people with an ABI can be exacerbated because of changes in their executive functioning and social cognition [39], which may make it challenging for them to recognize cyberscams or regulate their own interactions. The complexity of these issues is evidenced by people with a TBI who report having been on the receiving end of negative comments on the web as well as having been the perpetrator of cyberbullying [40]. Following the first 6 months of the COVID-19 pandemic when many countries underwent various levels of restrictions on movement and gatherings, the eSafety Commissioner (Australia’s national independent regulator for cybersafety) reported a noticeable increase in internet hate and harassment [41]. Recent work led by Gould et al [42] has also identified that injury-related cognitive impairments and social isolation increased the vulnerability of people with ABIs to cyberscams, particularly romance scams, and that the current lack of effective intervention may lead to scam revictimization. As such, it is imperative that resources and guidance are available to support the safe use of social media for connection, particularly for those who may be more vulnerable to web-based hate or scams through direct targeting from cyberscammers and trolls.

Even before the COVID-19 pandemic, it was reported that >60% of the general public used social media for everyday communication and socializing [43]. Thus, before acquiring a brain injury, it is highly likely that people will have developed a range of skills and competencies across various social media platforms and may want to return to using them in their daily interactions. Subsequently, during rehabilitation, clinicians should consider the person’s use of social media when examining communication contexts before their injury [29]. Rehabilitation clinicians have expressed willingness to support people in using social media after their brain injury [44]. However, they report uncertainty and concern regarding the potential risks people with a brain injury may encounter on social media [44]. Standard speech pathology clinical practice and ABI rehabilitation are yet to include social media skills training, and evidence-based guidance on social media use for brain injury rehabilitation professionals is lacking [33]. In Australia, despite growing recognition that people with disabilities are overrepresented in reporting negative social media experiences, there are limited social media cybersafety resources available that are accessible for adults with cognitive impairments. For example, at the time of writing, the eSafety Commissioner has made only 4 of their extensive range of resources available on their website in an *easy English* format [45]. With little access to guidance or support, rehabilitation professionals use greater caution in therapy, resulting in restrictive or reactive approaches rather than proactive interventions [44].

### Objective

To date, there has been one pioneering review of social media use by people with a TBI [28], indicating an urgent need for research in this area. Other reviews have demonstrated that peer support group interventions are a promising way to support individuals and promote adjustment following an ABI [46] and that digital health interventions can improve psychosocial and health outcomes for people with ABI [47]. Although peer support for people with ABI in online environments has been occurring with greater frequency, the efficacy of such support is yet to be explored. Recent studies have identified that, for people with TBI, similar barriers and facilitators affect both web-based and real-world social participation after their injury [48]. Although a previous review presented evidence for incorporating technology into cognitive-communication rehabilitation following TBI [49], no review has examined the evidence for training to support the use of social media generally, let alone for individuals who have cognitive-communication difficulties after a specific health condition such as an ABI. There is a pressing need to determine the outcomes and cost-effectiveness of social media training for people with an ABI [29]. As such, this review was developed to be intentionally broad, summarizing the literature across health conditions, population subgroups (ie, across the life span), and intervention strategies. It was also designed to incorporate user preference studies as well as freely accessible resources available to the general public, to elucidate what people with an ABI or their supporters may encounter when attempting to access support or guidance themselves through *Googling it*. The aims were well suited to a scoping review, which is ideal for reviewing a large and complex body of information across multiple sources not previously reviewed [50,51]. The specific research questions that guided this integrative scoping review are as follows:

1. What studies have investigated training for developing social media skills and safety?
2. What free web-based resources are used for social media skills training that are available to the general public?
3. What online support groups, aimed at providing peer connections and support, are available for people with an ABI?

## Methods

### Overview

An integrative scoping review was conducted to locate the following: (1) research relating to the use of training resources for developing social media skills and safety, with a focus on resources for people living with health conditions or their supporters (eg, rehabilitation professionals, family, friends, and direct support workers); (2) free web-based resources for social media skills training for the general public; and (3) online support groups for people with an ABI that are aimed at providing peer connections and support. The authors approached this scoping review with relativist ontological and constructivist epistemological positions [52], believing that meaning arises from interactions between individuals, their worlds, and their communities. As such, a pragmatic approach was applied [53], using mixed methods to understand the common and distinct components of social media skills training for people with an ABI. The review focused only on currently available web-based resources and support communities and used the PRISMA-ScR (Preferred Reporting Items for Systematic Reviews and Meta-Analyses extension for Scoping Reviews) guidelines [54]. A review protocol was written before commencing this study (Multimedia Appendix 1 [55-65]), with systematic searching initially conducted in March 2020 (by the first author [MB] and a student intern) and a secondary search conducted in November 2020 (by the first author, MB). The research team comprised 3 qualified speech pathologists who were also experienced researchers in the fields of ABI and digital health and a student intern. The first (MB) and second (RR) authors conducted data extraction in conjunction with the student. Multimedia Appendix 2 [54] provides the completed PRISMA-ScR checklist for this scoping review.

Each of the 3 research questions was explored using a specific approach for the search strategy, exclusion criteria, study selection, data extraction, and critical appraisal. The specific details of each approach are provided in Multimedia Appendix 1.

### Search Strategy and Study Selection

#### Peer-reviewed Academic Literature Investigating Social Media Training

To identify relevant evidence, we included any publication in a peer-reviewed journal in the English language relating to the use of training resources for social media skills training. Resources for any population were included, although the search strategy was targeted toward people living with health conditions or their supporters. This included both descriptions of the development of such resources and reporting of the outcomes of the use of the resources, as well as user perspectives on such resources or training needs in the use of social media. Training resources were defined as any written content (including both webpages and downloadable PDFs), training packages (websites or apps), or audiovisual content. The systematic searching and selection based on title and abstract were conducted by the first author (MB), with selection based on full-text articles reviewed independently by the first (MB) and second (RR) authors and discrepancies resolved through discussion to achieve consensus.

#### Free Web-Based Social Media Training Resources

To identify currently available free web-based resources, we included any web-based resource targeted at the general public that was aimed at improving social media skills. The authors defined web-based resources as any written content (including both webpages and downloadable PDFs), training packages (websites or apps), or audiovisual content available via the internet, as identified through Google searching.

#### Online Support Groups for People With an ABI

To identify the currently available online support groups for people with a brain injury, we included any existing web-based community targeted at people with an ABI (and their supporters, such as family and friends) that was aimed at providing peer connections and support. The authors defined web-based communities as any networking groups or communities available via the internet, as identified through networks known to the researchers, Google searching, and Facebook searching.

### Data Extraction, Critical Appraisal, and Synthesis

Data were extracted from all information sources and managed using Microsoft Excel spreadsheets [55] that were accessed by the research team to ensure consistency and transparency. Specific details regarding the data extraction, critical appraisal, and data synthesis processes used across the 3 information sources are presented in Multimedia Appendix 1. Data were synthesized descriptively to map different aspects of the literature and the resources outlined in our key questions. Descriptive statistics were calculated using Microsoft Excel [55]. The data collected from the charting process and critical appraisal were compared and synthesized qualitatively using open coding [66] across information sources to identify similarities in the format and content of web-based social media training and support resources. An inductive approach to content analysis was used, in which codes and categories were derived from the data, to enable the description and categorization of the data without potentially restricting the findings [67]. The authors discussed the findings in the context of ABI rehabilitation, and a constant comparison was used to compare and integrate the data [68]. This allowed the research team to identify key issues to address and features to use in social media training for people with an ABI [53] to inform future resource development and implementation research.

## Results

### Overview

**Figure 1:**
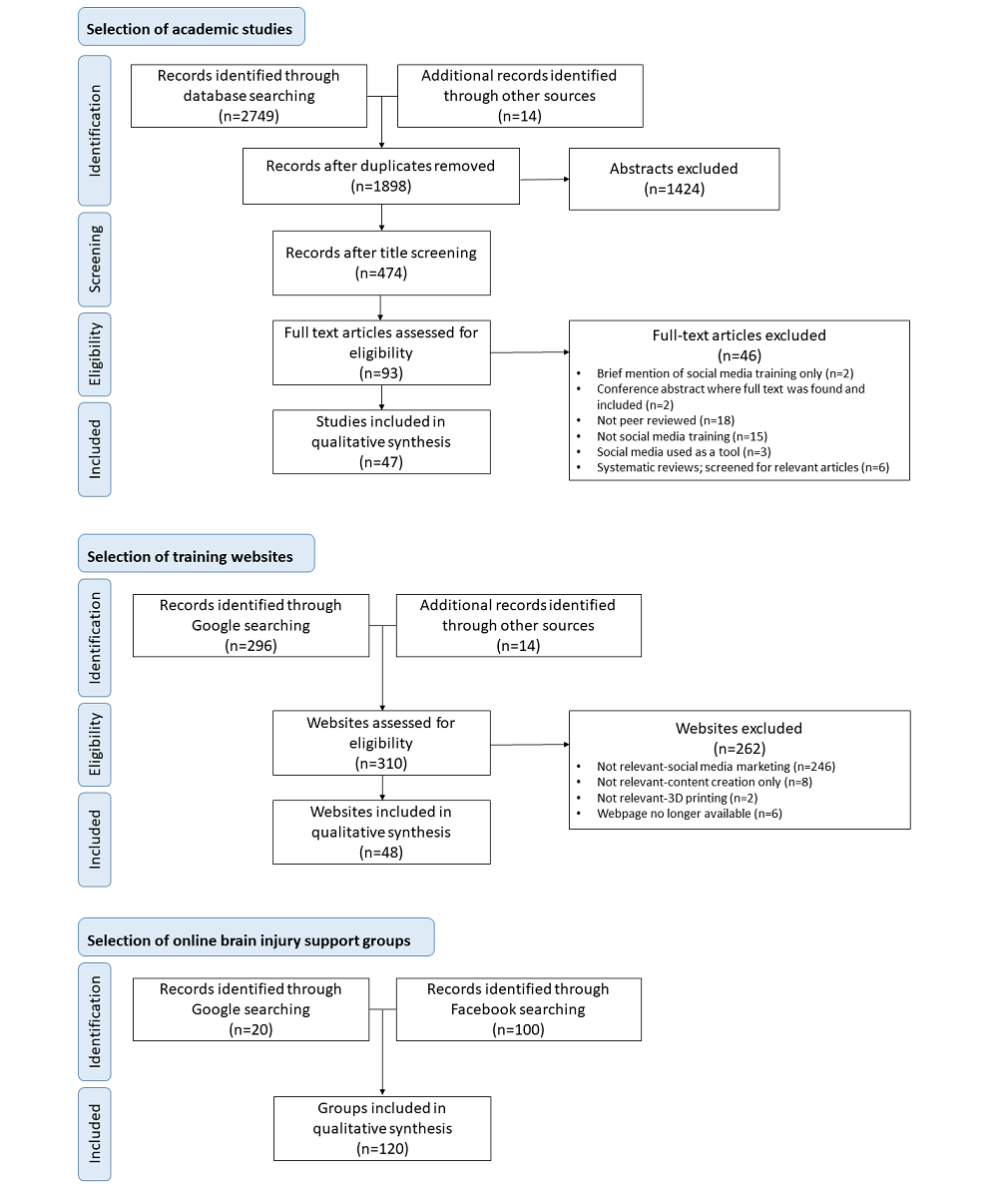
The flow of information sources through the inclusion process [54].

A total of 47 peer-reviewed articles, 48 social media training websites, and 120 online support groups were included in this review. Figure 1 outlines the flow of sources through the inclusion process [54], and Table 1 provides a description of the target populations and behaviors identified across the 3 information sources). The completed PRISMA-ScR checklist can be found in Multimedia Appendix 2.

**Table 1:** Target populations and behaviors identified across the 3 information sources.

		Academic literature (N=47), n (%)	Social media training websites (N=48), n (%)	Online support groups (N=120), n (%)
**Target population or audience**				
	Neurotypical	21 (44.7)	47 (97.9)	15 (12.5)
	Cognitive disability	22 (46.8)	1 (2.1)	105 (87.5)
	Physical disability	6 (12.8)	N/A^a^	N/A
	Communication disability	11 (23.4)	N/A	N/A
	Adults	35 (74.5)	46 (95.8)	120 (100)
	Children (aged <13 years) and young people (aged 13-18 years)	14 (29.8)	2 (4.2)	N/A
**Target behaviors**				
	Social participation	17 (36.2)	N/A	120 (100)
	Professional use (eg, for employment or social marketing)	10 (21.3)	8 (16.7)	N/A
	Cybersafety	7 (14.9)	11 (22.9)	N/A
	Social media, information and communication technology knowledge, or use	8 (17)	15 (31.2)	N/A
	Content creation	N/A	8 (16.7)	N/A
	Well-being or other (mental health, sleep, eating, life skills, and support)	5 (10.6)	6 (12.5)	120 (100)

^a^N/A: not applicable.

### Question 1: Peer-reviewed Academic Literature Investigating Social Media Training

Of the 47 included articles [37,69-114], most studies were conducted in either the United States (20/47, 43%) or Australia (20/47, 38%), with a median publication date of 2017 (range 2011-2020). Nearly two-thirds of the included studies were published as journal articles (30/47, 64%), with the remainder published as conference proceedings (13/47, 28%) or conference abstracts (4/47, 9%). These studies used a diverse range of qualitative (19/47, 40%), quantitative (15/47, 32%), and mixed methods (13/47, 28%) research designs. The target populations involved were also diverse but were identified as being vulnerable in some way when using social media. For example, there were studies with young people exploring cyberbullying [76,94,97,106] and studies with older adults working on building their internet and social media confidence [69,70,87]. Of the 47 included studies, 22 (47%) included people who had a cognitive disability, 21 (45%) included *neurotypical* individuals, and 11 (23%) specifically investigated social media training for people with communication disabilities. Behaviors of interest included social participation (17/47, 36%), professional use of social media (10/47, 21%), generic social media or internet knowledge and use (8/47, 17%), cybersafety (7/47, 15%), well-being (3/47, 6%), or life skills (2/47, 4%). Most intervention studies involved training delivered in person (25/33, 76%) and on the web (9/33, 27%).

The methodological reporting in the articles included in this review was variable in quality. A total of 13% (6/47) of the articles were conference abstracts [78,92,95,99,103,108] with limited study details where the methodological rigor could not be appraised. Of the 41 articles, 26 (63%) appraised were considered to have reported either strong methodology (a rating score >80% of the specified tool criterion) [37,69,70,75,77,80,83,86,88,91,93,94,97,100-102,106,112] or moderately strong methodology (a rating score >50% of the specified tool criterion) [72,74,76,79,87,89,96,104]. Multimedia Appendix 3 [37,69-114] provides a description and critical appraisal scores for the included studies.

### Question 2: Free Web-Based Social Media Training Resources

Of the 48 included websites (Multimedia Appendix 4), 46 (96%) were directed toward *all ages* or an adult audience, with only 2 (4%) websites tailored for children or young people. Of the 48 websites, 9 (19%) were targeted toward teachers and parents, and 3 (6%) catered for more niche populations, specifically women, older people, and employees. Of these 48 websites, only 1 (2%) considered neurodiversity at all [115] and highlighted the risk that people with learning disabilities may encounter when using social media. Two-thirds of the training websites offered text-based resources (32/48, 67%), with the remainder offering courses and workshops (9/48, 19%) or videos and podcasts (7/48, 15%) and targeted generic *How to use social media* skills (15/48, 31%), cybersafety (11/48, 23%), social marketing or employment (8/48, 17%), content creation (8/48, 17%), or well-being (6/48, 13%).

The web accessibility and readability of the included social media training websites were evaluated to determine their suitability for someone who has difficulties with cognition or communication that can commonly occur after a brain injury.

*Web accessibility* is defined as the websites, tools, and technologies that are designed and developed so that people with disabilities can use and navigate the internet [116]. When websites are poorly designed, they can create barriers, often preventing people from using them as intended. The Web Content Accessibility Guidelines (WCAG) 2.1 [117] provide recommendations on how to make website content more accessible to a wider range of people with disabilities, such as visual, auditory, physical, speech, cognitive, language, learning, and neurological disabilities, “but will not address every user need for people with these disabilities” [117]. The WCAG 2.1 has 3 conformance levels, of which the highest is Level AAA, indicating that a webpage satisfies all the recommended criteria for making it as accessible as possible [117]. Of the 48 included websites, none conformed to Level AAA, with only 1 (2%) website [118] identified as having less than 10 Level AAA accessibility issues. Most of the websites (30/48, 63%) were found to have >20 Level AAA conformance issues identified, indicating that these websites are likely to be difficult for people with a brain injury to use and navigate.

*Readability* refers to how easily a person can read and understand written text [119]. Organizations such as the American Medical Association and the Agency for Healthcare Research and Quality recommend that the readability of health information materials should not be greater than a sixth-grade reading level [120,121]. In the Australian context, it is also generally recommended that health information be presented using plain language and should aim for a reading grade level of 6 (neurotypical children aged 11-12 years who have English as their first language; eg, [122-125]). More recently, it has been recommended that when preparing digital content, “you write your content for a reading level of age 9 or lower. If you’re unable to achieve reading age 9, the more readable you can make your content, the better” [126]. Most included social media training websites had a reading grade level of 7 or above (39/48, 81%), a level considered high for the general public and indicating that these websites are highly likely to be difficult for people with a brain injury to read and understand. Table 2 provides an overview of the included websites, and Multimedia Appendix 3 provides a list of the included websites.

**Table 2:** Characteristics, accessibility, and readability of the web-based social media training websites (N=48).

			Value, n (%)
Text based			32 (67)
Courses or workshops			9 (19)
Videos or podcasts			7 (15)
**Accessibility**			
	Conformance to international WCAG^a^ 2.1 (Level AAA)	0 (100)	
	<10 issues^b^ identified	1 (2)	
	<20 issues identified	18 (38)	
	≥20 issues identified	30 (63)	
**Readability**			
	Flesch-Kincaid reading grade level 6 or below^c^	9 (19)	
	Flesch-Kincaid reading grade level 7 or above	39 (81)	

^a^WCAG: Web Content Accessibility Guidelines.

^b^Occurrences of an issue (error, warning, or review item) determined to be in contravention of the WCAG 2.1.

^c^Flesch-Kincaid reading grade level 6 or below is easily understood by neurotypical children aged 11 to 12 years who have English as their first language.

### Question 3: Online Support Groups for People With an ABI

Of the 120 included online support groups, 102 (85%) were Facebook groups or pages, 16 (13.3%) were web-based discussion forums, and 2 (1.7%) offered support meetings via web-based videoconferencing. Of the 120 groups, 62 (51.7%) of the groups did not specify a location or were branded as being global. Of those that specified a location, the majority were based in the United States (38/120, 31.7%), with a smaller number of groups located in Australia (13/120, 10.8%), Canada (4/120, 3.3%), Aotearoa or New Zealand (2/120, 1.7%), the United Kingdom (2/120, 1.7%), and India (1/120, 0.8%). All groups aimed to provide social participation and support for members. Most of the groups catered to people with a brain injury (102/120, 85%), with 12.5% (15/120) of the groups targeted toward family and caregivers of people with a brain injury, and 2.5% (3/120) of the groups catering specifically to women with a brain injury. Most of the groups were closed (private) or required users to request permission to join the group (97/120, 80.8%), with a smaller number of groups using an open format (23/120, 19.2%), where information on membership and posts are available and easily accessible to the general public. Many of the groups were moderated by people with an ABI (20/120, 16.7%), family or caregivers (4/120, 3.3%), or brain injury support organizations (47/120, 39.2%). However, for 40.8% (49/120) of the groups, it was unclear from the public group description as to who managed the group, although several implied that it may be organized by someone with a brain injury, a family member, or a health professional. None of the support group *about* statements made any direct reference or inference to social media skills training. Table 3 provides an overview of the included online support groups, and Multimedia Appendix 5 provides a list of the included support groups.

**Table 3:** Characteristics of the online social media brain injury support groups (N=120).

			Value*,* n (%)
**Type of platform**			
	Facebook group or page	102 (85)	
	Web-based discussion forum or space	16 (13.3)	
	Online support meetings (using web-based videoconferencing)	2 (1.7)	
**Type of space**			
	Open or public group	23 (19.2)	
	Closed or need to request to join the group	97 (80.8)	
**Target audience**			
	People with a brain injury (adults)	102 (85)	
	Women with a brain injury (adults)	3 (2.5)	
	Family or caregivers of people with a brain injury (adults and children)	15 (12.5)	

### Integration of Findings

Across the 3 information sources, key issues relating to the content and format of social media training were identified and synthesized. The topics of how to use social media and cybersafety were present across all 3 information sources. Other frequent topics identified were the following: developing relationships in social media, how to use technology to access social media, maintaining relationships, finding support for people who can guide social media use and troubleshooting, how to connect and interact with peers, navigating personal and professional use of social media, being able to access technology through assistive devices, and how to improve or maintain your well-being. Similarities in the training format were less apparent across the data sources. However, all the sources included training or guidance that incorporated interactive elements, allowing a two-way flow of information. In addition, the following features were identified in some of the data sources: led by people with an ABI, offer choice to cater to individual preferences, provide opportunities for real-life practice and where possible include practical examples, provide support for memory and recall, and, when able, tailor training to the individual’s needs. Table 4 outlines in which of the 3 information sources these issues were identified.

**Table 4:** Key issues to address in social media training (or resources) identified across the 3 information sources.

Key issues			Academic literature		Social media training websites		Online support groups
**Content (topics)**							
	Developing relationships	✓				✓	
	How to use technology	✓					
	How to use social media	✓		✓		✓	
	Maintaining relationships	✓					
	Cybersafety	✓		✓		✓	
	Support people	✓				✓	
	Peer connection	✓				✓	
	Personal and professional use			✓			
	Technology access	✓					
	Well-being			✓		✓	
**Format (features)**							
	Interactive	✓		✓		✓	
	Led by and designed with people with an acquired brain injury	✓				✓	
	Offer choice	✓				✓	
	Opportunity for real-life practice	✓				✓	
	Practical examples	✓		✓			
	Support memory and recall	✓					
	Individually tailored	✓					

## Discussion

### Principal Findings

In this review, we identified 47 articles that explored social media skills training across diverse populations, which provided information to support the future development of training for people with an ABI. Across the 48 websites that were freely accessible to the public, we found considerable variability in the content, readability, and accessibility of the information provided regarding social media skills training. For many of the 130 online support groups identified, it was unclear who managed the group, and none directly referenced social media skills training. Our synthesis of the data across the 3 information sources (peer-reviewed literature, websites, and online support groups) identified several key issues that are critical to addressing social media training for people with a brain injury. Evidence regarding the effectiveness of social media training was limited, and the authors were unable to draw robust conclusions about which active components of content and techniques for delivery were more successful than others. However, it was evident that training programs would best be engaging and interactive, developed using a co-design process by collaborating with people with an ABI and their supporters (eg, rehabilitation clinicians, family, friends, and industry stakeholders). This corroborates previous work that identified the need for specific research involving the user-centered co-design, development, and evaluation of web-based social media training for people with a brain injury [29]. It is also vital that training resources meet the needs of ABI rehabilitation professionals to facilitate the adoption of social media use as part of their rehabilitation intervention services [44].

Furthermore, the findings of this review indicated that the content of social media skills training should primarily provide general information about how to use social media and how to stay safe. In addition, training should demonstrate how to communicate and connect with others on key social media platforms (ie, Facebook, Twitter, and Instagram). Information should be presented using techniques that support recall and retention [85,87,110], along with support strategies for executive function (eg, strategies that promote learning or everyday problem solving) [90,101,111]. The importance of real-life practice that is personally meaningful [127-129] and being able to practice with everyday communication partners [128,130] has been well established in evidence-based cognitive rehabilitation programs for people with an ABI. Therefore, opportunities to practice key navigational, communication, and safety skills for using social media are likely to be critical components of successful training programs. Similarly, training programs that can be tailored to the individual, or at least provide users the ability to self-select which modules are completed and in which order they are completed, would be optimal. As few of the included websites met the recommended criteria for readability and accessibility of their information, we suggest that when developing web-based resources, it is imperative that the readability of the materials be appropriate for the general public (ie, below reading grade 6 level) and the information accessible (ie, comply with WCAG 2.1 recommendations). In combination with priorities for learning about social media from interviews with people with a TBI [37,40] and brain injury rehabilitation professionals [44], the findings of this review can now be used to inform user-centered co-design discussions regarding the development of social media skills training programs for people with an ABI. In an interim step, clinicians may use the findings (specifically those outlined in Table 4) as guidance in clinical practice in providing social media training to support people with an ABI after their injury.

Regarding the online support groups available for people with a brain injury (and their supporters), there were an overwhelming number of groups, many moderated by people with an ABI or brain injury support organizations. However, for many others, it was unclear who ran the group. Most groups were closed or private, indicating a user preference for increased privacy. This may be due to the knowledge that open groups can leave people vulnerable to broader public commentary and being targeted by cyberscammers or trolls. Although closed groups provide users with some privacy, it should be noted that until joining the group, a user is unable to determine the membership of the group or the content of the group’s posts. This may mean that users are faced with difficult decisions regarding which group to join. Findings from this review may facilitate clinicians’ identification of relevant websites or online support groups to suggest to families of people with an ABI to support social media skills training and guidance.

### Limitations and Directions for Future Research

This review had several limitations. First, in the interest of timeliness, the first author (MB) conducted the data extraction and critical appraisal with a student intern, and a second academic reviewer was not used. Second, although we used a systematic and comprehensive search strategy, some relevant websites and online support groups may have been omitted. Given the ephemeral nature of the internet, additional social media training website resources and online support groups for people with a brain injury may have been developed since the searches were conducted. Indeed, since this review was conducted, there have been websites that have been developed to address cybersafety and social media use for people with an ABI, such as the CyberABIlity [131] and social-ABI-lity [132] training programs. Similarly, the design and content of the included websites and online support groups are likely to change over time. In addition, as the evaluation of the included online support groups was beyond the scope of this review, the benefits and risks of these groups warrant further investigation. Therefore, clinicians using Multimedia Appendices 4 and 5 provided in this review (ie, the lists of websites and online support groups) should confirm the presence and credibility of any website or support group before referring individuals to it.

The findings of this research also suggest that, alongside research exploring the development and implementation of co-designed social media skills training resources for people with a brain injury and their supporters, greater insights into the experiences and perspectives of rehabilitation professionals are required, in addition to studies exploring cybersafety and web-based self-representation of people with an ABI on social media. Robust research is urgently needed to evaluate the effectiveness of the potential key ingredients for the content and delivery of social media training resources for people with an ABI that were identified in this scoping review and have been implemented in web-based training resources such as CyberABIlity [131] and social-ABI-lity [132]. Further research is needed to determine how using social media following an ABI may support social participation, cyber-resilience, and growth in personal agency. The results of such studies likely have the potential to inform resources adaptable for other people who experience difficulties in social communication, for example, young people with developmental language disabilities or adults with dementia.

### Conclusions

There is limited research exploring social media skills training, with few web-based training resources available for people with an ABI. Social media offers an important means of connection for people with an ABI, providing continual opportunities for them to observe and interact with others, as well as a way to develop new relationships when their cognitive-communication skills may limit their in-person interactions. The findings from this review, along with priorities for learning about social media informed by people with a brain injury and rehabilitation professionals, can be used to inform the development of novel web-based training resources to support social media skill development in people with an ABI. The development of such resources may drive sustainable change through the provision of clinical practice guidance and the creation of web-based support networks to help people with a brain injury build their own social media mastery.

## Appendix

Multimedia Appendix 1 Scoping review protocol for the search strategy, exclusion criteria, study selection, data extraction, and critical appraisal applied across the 3 information sources.

Multimedia Appendix 2 PRISMA-ScR (Preferred Reporting Items for Systematic Reviews and Meta-Analyses extension for Scoping Reviews) checklist.

Multimedia Appendix 3 Included academic peer-reviewed literature (N=47).

Multimedia Appendix 4 List of included websites.

Multimedia Appendix 5 List of included online support groups.

## Abbreviations

ABI: acquired brain injury
PRISMA-ScR: Preferred Reporting Items for Systematic Reviews and Meta-Analyses extension for Scoping Reviews
TBI: traumatic brain injury
WCAG: Web Content Accessibility Guidelines
