# Supplementary material for: Training Resources Targeting Social Media Skills to Inform Rehabilitation for People Who Have an Acquired Brain Injury: Scoping Review
Source: J Med Internet Res. 2022 Apr 28;24(4):e35595. doi: 10.2196/35595 (PMC9100544; doi:10.2196/35595)
Supplement: Multimedia Appendix 1 [file jmir_v24i4e35595_app1.docx]

Multimedia Appendix 1. Scoping Review Protocol for the Search Strategy, Exclusion Criteria, Study Selection, Data Extraction, and Critical Appraisal applied across the Three Information Sources

|  | **Question 1:**  **Peer-reviewed academic literature investigating social media training** | **Question 2:**  **Free online social media training resources** | **Question 3:**  **Online support groups for people with acquired brain injury (ABI)** |
| --- | --- | --- | --- |
| Exclusion Criteria | (a) Does not relate to training or user-perspectives on social media use or safety  (b) Provides a brief recommendation or overview only (including conference abstracts without additional information)  (c) Uses social media as a tool for intervention/recruitment/training only  (d) Not published in English  (e) Not published in a peer-reviewed journal or an accepted doctoral thesis  (f) Conference abstract only, full publication available  (g) Published before 2010 | (a) Does not relate to improving personal social media skills or safety  (b) Not targeted at the general public (i.e., academic or specialist professional audience)  (c) Not available free of charge  (d) Not in English  (e) Information is specific to improving social media marketing skills | (a) Does not provide connections with other users  (b) Does not relate to people with ABI  (c) Not targeted at the general public (i.e., academic or specialist professional audience)  (d) Not available free of charge  (e) Not in English |
| Information Sources | OvidSP (Medline and AMED and PsycINFO and Embase), Scopus, Web of Science, CINAHL, and Google Scholar | Google | Google and Facebook |
| Search Strategy | (“training” OR “education” OR “intervention” OR “information” OR “resources” OR “support”) AND (“Internet” OR “online” OR “on-line” OR “web-based”) AND (“social media” OR “social networking site” OR “Facebook” OR “Twitter” OR “blog” OR “Instagram” OR “YouTube” OR “SnapChat” OR “Reddit” OR “LinkedIn”) AND (“health” or “healthcare” or “illness” or “disability”) | A series of Google searches (using the setting of “any country”) was executed using the starting point of generic search terminologies which could be used to search Google to seek help with using social media (“How can I learn social media?”, "online social media training free", "free course how to use social media", "social media tutorials for beginners"). Related search terms suggested by Google which were also relevant to the topic of this scoping review were then used as follow-up searches, with further relevant search terms identified using an iterative process. | A series of Google searches (using the setting of “any country”) was executed using the starting point of a generic search term which could be used to search Google to find online peer support communities for people with acquired brain injury and their supporters (“online support groups for people with brain injury”, "online support groups ABI", "social media people with brain injury"…). Related search terms suggested by Google which are also relevant to the topic were then used as follow-up searches, with further relevant search terms identified using an iterative process. Another series of searches within Facebook was conducted using the search terms: ABI, TBI, and brain injury support. |
| Selection of sources of evidence | The database searches were compiled and duplicates removed in Endnote [56]. Initial screening (first author) removed any results which did not meet the criteria based on the title and abstract. The full-text articles were reviewed independently (first and second author) for the remaining results, with discrepancies resolved through consensus discussion. The citations of the included full text articles were then transferred to an Excel datasheet [55]. | For each of the Google searches, the URLs of the first 100 search results were noted (first author). The list of search results were combined and duplicates removed using an Excel datasheet [55]. Initial screening removed results identified as not meeting the inclusion criteria based on the title and the remaining web pages reviewed in full to identify relevant resources (student intern). During the review of full web pages, any links to further web pages with potentially relevant online resources were noted (student intern and first author). These web pages were screened against the inclusion criteria for potential inclusion in the review (first author). | For each of the Google searches, the URLs of the first 100 search results were noted (first author). The list of search results were combined and duplicates removed using an Excel datasheet [55]. Initial screening removed results identified as not meeting the inclusion criteria based on the title and the remaining web pages reviewed in full to identify relevant resources (student intern and first author). During the review of full web pages, any links to further web pages with potentially relevant online resources were noted. These web pages were screened against the inclusion criteria for potential inclusion in the review (first author). |
| Data Extraction | Data was extracted relating to each online resource using an Excel datasheet [55] developed for the purpose of this scoping review (first author). The online resources were described based on the following variables: (a) publishing organisation or author, (b) year of publication, (c) intended audience (i.e., paid or unpaid carers), (d) format (i.e., text, audio, video, interactive components), (e) length or duration (i.e., number of words or duration of video / training course), (f) theoretical basis, (g) any aspects of tailoring of information to the type of communication partner or specific needs of the individual with TBI, and (h) any evidence supporting the use of the resource. | Data was extracted relating to each online resource using an Excel datasheet [55] developed for the purpose of this scoping review (student intern and first author). The online resources were described based on the following variables: (a) publishing organisation or author, (b) year of publication, (c) intended audience (i.e., paid or unpaid carers), (d) format (i.e., text, audio, video, interactive components), (e) length or duration (i.e., number of words or duration of video / training course), (f) theoretical basis, (g) any aspects of tailoring of information to the type of communication partner or specific needs of the individual with TBI, and (h) any evidence supporting the use of the resource. | Data was extracted relating to each online resource identified using an Excel datasheet [55] developed for the purpose of this scoping review (student intern and first author). The online resources were described based on the following variables: (a) Founder/Supporting organisation, (b) Target audience, (c) Type of Platform, (d) How to join, (e) Moderation and safety policies, (f) Aim of the group/community space, (g) Location, (h) Cost. |
| Critical appraisal of individual sources | Peer-reviewed publications were appraised using the PEDro-P Scale for Critical Appraisal of Group Comparison Studies [57] for controlled trials; the Joanna Briggs Institute (JBI) Checklist for Quasi-Experimental Studies [58]; the JBI Checklist for Cohort Studies [59]; the Center for Evidence-Based Management Critical Appraisal of a Survey checklist for survey studies [60]; the JBI Checklist for Qualitative Research [61]; and the Mixed Methods Appraisal Tool [62]. Conference abstracts with limited information were not appraised. The first author conducted the appraisals. | The online resources were appraised using the SiteImprove Accessibility Checker [63] and the WebFX Readability Test Tool [64] or the Microsoft Word Readability Statistics [65], which provided automated scores relating to the accessibility and readability of the resource. The student intern conducted the readability ratings, and the first author conducted the accessibility ratings and confirmed readability ratings. | Not appraised. |
