# Supplementary material for: Training Resources Targeting Social Media Skills to Inform Rehabilitation for People Who Have an Acquired Brain Injury: Scoping Review
Source: J Med Internet Res. 2022 Apr 28;24(4):e35595. doi: 10.2196/35595 (PMC9100544; doi:10.2196/35595)
Supplement: Multimedia Appendix 3 [file jmir_v24i4e35595_app3.docx]

Multimedia Appendix 3. Included academic peer-reviewed literature (N = 47).

| First author  [Reference] | Year | Study design | Social media platform/s | Country | Population | Intervention | Target Behaviour | Critical Appraisal |
| --- | --- | --- | --- | --- | --- | --- | --- | --- |
| Arthanat [69] | 2016 | Quant | ICT - included Facebook, skype, email | United States of America | 13 older adults (62-83 yrs) with no cognitive impairment or support needs | A 3 month, in-person ‘personalised coaching’ training program | ICT skills | JBI QE 7/9 |
| Arthanat [70] | 2019 | Qual | ICT - included Facebook, skype, email | United States of America | 61 participants in three stakeholder groups: older-adult ICT trainees, care providers and ICT trainers | As per Arthanat 2016 study | ICT skills | JBI Qual 8/10 |
| Barber  [71] | 2017 | Quant | ICT - included 'social media' | United States of America | 78 university students | An automated PowerPoint presentation (STEPS-TECH, 22 minutes and 33 seconds long) | Sleep | PEDro-P 5/11 |
| Bayor  [72] | 2018 | Qual (Protocol) | YouTube, Facebook | Australia | Young adults with an ID aged 18-34years, in an Australian DSO setting | In-person training & co-design workshops | Social life skills | JBI Qual 6/10 |
| Bayor  [73] | 2019 | Qual | Facebook, YouTube, Snapchat, Instagram | Australia | 10 young adults with ID (four males and six females) aged between 18-34 years, receiving literacy and social inclusion support services from a partner DSO. | In-person training & co-design workshops “TechShops” | Skills development | JBI Qual 2/10 |
| Bayor  [74] | 2019 | Qual | YouTube, Facebook | Australia | 11 young adults with ID (six males and five females aged 18-34 years) receiving services at the DSO. | In-person training & co-design workshops “TechShops” (1½ hours per session, twice weekly, plus take-home exercises, and posting videos on a Facebook group) | Skills (of interest) development | JBI Qual 7/10 |
| Bramstedt  [75] | 2014 | Quant | Social media | Australia | 167 fourth- and fifthyear medical students completed the compulsory session (82 students in Year 4 and 85 students in Year 5) | A compulsory online session in social media ethics (22 minutes duration, delivered in two online modes: a narrated PowerPoint file and a YouTube video) | Knowledge of using social media for information-sharing and issues around professionalism | JBI C 7/11 |
| Brunner  [37] | 2019 | Qual | Social media | Australia | 13 adults (seven men, six women) with TBI and cognitive-communication disability | N/A | Social participation and communication | JBI Qual 8/10 |
| Cardoso  [76] | 2020 | Qual | Social media (e.g., Instagram, Facebook, YouTube) | Australia | 68 young people (57% female, 43% male), aged between 13 and 15 years (and their parents) | N/A | Safe electronic image sharing | JBI Qual 5/10 |
| Caron  [77] | 2017 | Qual | Social media (Facebook, Twitter, Instagram, Apple messages, Snapchat, Vine) | United States of America | 7 adolescents and young adults with cerebral palsy who all used high-tech speech generating devices to communicate (four females and three males, ranging in age from 14– 21) | N/A | Social participation and communication | JBI Qual 8/10 |
| Caton  [78] | 2019 | Qual | Social media (Instagram, Facebook, Snapchat, Twitter, Messenger, Musical.ly, WhatsApp, Xbox, PlayStation, Skype, YouTube, Discord, Steam, Twitch, Reddit, Ebay) | United Kingdom | 31 young people with intellectual disabilities (aged 11-20) | An 11-month, user-led awareness and training project - the Get SMART (Social Media Awareness and Resilience Training) project | Knowledge of internet safety, online radicalisation and grooming for terrorism | CA |
| Daems  [79] | 2015 | Qual | Social media (Facebook, Skype, WhatsApp, Twitter, Instragram) | Belgium | 23 people with IDD | N/A | Knowledge of social media | JBI Qual 6/10 |
| Flickinger  [80] | 2015 | Mixed | Social media (SNSs, information sharing sites, blogs, micro-blogs) | United States of America | 91 medical students (survey), 11 med students (social media pilot) | Two 90-minute workshops, with 60 students each; a private, chaperoned blog. | Knowledge of using social media for information-sharing and issues around professionalism | MMAT 13/17 |
| George  [81] | 2011 | Mixed | Social media (Facebook, Twitter, LinkedIN, Blogs, RSS readers, Google resources) | United States of America | 15 health professionals | Three 60-minute in-person “Friending Facebook?” course lessons | Knowledge of using social media as a resource and for information-sharing and issues around professionalism | MMAT 2/17 |
| Gilster  [82] | 2020 | Mixed | Social media (Twitter, LinkedIn, Instagram, Facebook, or YouTube) | United States of America | 25 social work students | In-class discussions focused on social media ethics, creating a professional presence, and aspects of successful social media use; Three video tutorials about social media; and a social media advocacy assignment (practical use of a platform) | Social media advocacy skills | MMAT 2/17 |
| Grace  [83] | 2014 | Quant | Social media (Skype, Facebook, Twitter) | Australia | 5 youth between the ages of 10–18 with complex communication needs who are unable to use speech for everyday communication and require augmentative and alternative communication | In-person training and provision of individualised guidelines for cybersafety; strategies and supports provided to participants and their families; phone support provided between visits | Internet and social media skills for social participation | JBI QE 7/9 |
| Gruba  [84] | 2013 | Quant | Social media | Australia | 53 AusAID scholarship students | Two 2-hour social media training workshops; then offered three 60-minute drop-in sessions for individual consultation | Social media use | CEBM CAS 0/12 |
| Hafez  [85] | 2018 | Quant | Facebook | United States of America | Protocol - mild cognitively-impaired elderly, aging between 65–80 years old | An introductory 50-minute practical social media class (including use of a mobile tutoring app prototype); a follow up class after two weeks to repeat the same previously performed activities | Social media use | PEDro-P 1/11 |
| Hafez  [86] | 2019 | Quant | Facebook | United States of America | 5 mild cognitively-impaired elderly adults age 65 and 70 years | A one-hour classroom training session, followed by a two-week self-study period (with Seniorbook, a mobile training app prototype, designed to resemble the activities frequently done in popular social media apps) | Social media use | JBI QE 6/9 |
| Harvey  [87] | 2019 | Qual | Twitter | United States of America | 9 'normally aging' older adults and 15 student SLPs | Thirteen 1-hr Twitter training sessions; and a social media engagement phase provided support in Twitter | Twitter use | JBI Qual 5/10 |
| Hemsley  [88] | 2018 | Mixed | Twitter | Australia | 3 people who use AAC - no cognitive impairment | One individual 2-hour tutorial on the strategic use of Twitter, delivered via Skype; a self-directed online training program (PowerPoint slideshow saved as a PDF) was emailed to participants one week prior to tutorial | Twitter use for social participation | MMAT 16/17 |
| Henning  [89] | 2017 | Qual | Facebook | Aotearoa/New Zealand, Australia, and Wales | 57 nursing, medical and paramedical students | N/A | Professional Facebook use | JBI Qual 7/10 |
| Holmes  [90] | 2014 | Qual | Facebook | United Kingdom | 3 people with learning disabilities | N/A | Social identity and participation | JBI Qual 2/10 |
| Kydland  [91] | 2012 | Mixed | Flickr | Norway | 12 people with intellectual disability (20 to 56 years old) employed at a rehabilitation company | An 8-week program providing a weekly, in-person practical guidance session; a closed Flickr group | Flickr use for social participation | MMAT 16/17 |
| Lellis-Santos  [92] | 2018 | Qual | Social media (Twitter) | Brazil | 39 adults attending a satellite meeting of the 38th IUPS World Congress held in Rio de Janeiro | One practical Twitter training workshop | N/A | CA |
| Maben  [93] | 2017 | Quant | Social media | United States of America | Advisers (n=112) and student leaders (n=31) from North American collegiate student-run communication organizations | N/A | Social media use as professionals | MMAT 14/17 |
| McLean  [94] | 2017 | Quant | Social media | Australia | 101 adolescent girls (Mean age 13.13, SD 0.33) recruited from one co-educational public and one private girls’ secondary school in Melbourne, Australia. | Three 50-minute experiential and interactive lessons “The Boost Body Confidence and Social Media Savvy (Boost)” delivered weekly | Eating disorder risk factors | JBI QE 8/9 |
| Menger  [95] | 2017 | Qual | Social media (Facebook, online messaging, email) | United Kingdom | 4 people with aphasia | Tailored interventions which consisted of 1 or more of: therapy targeting impairment of language or non-verbal cognition, compensatory strategies, assistive technology, modification of online environments, and provision of one to one support. | Internet use and skills for communication and leisure | CA |
| Mentis  [96] | 2019 | Qual | Social media | United States of America | 6 couples - older adults with mild cognitive impairment (MCI) and their spousal caregivers | N/A | Cybersecurity | JBI Qual 7/10 |
| Prihastuty  [97] | 2019 | Quant | Social media | Indonesia | 30 students who are members of the OSIS (Organisasi Siswa Intra Sekolah) aged 15 to 17 years | One day-long social media literacy training program | Cyberbullying | JBI QE 7/9 |
| Probst  [98] | 2017 | Qual | Social media (Instagram) | United States of America | 16-year-old female student diagnosed with an autism spectrum disorder, ADHD and anxiety | One in-person interview (questions/prompts for critical understanding of media production and consumption) | social participation | JBI Qual 4/10 |
| Raghavendra  [99] | 2016 | Mixed | Social media | Australia | 7 young people with developmental disabilities as mentees, and two mentors. Mentees were aged between 12.2 and 19.11 years (Mean=16.7y, SD=2; 4y), had developmental disabilities and lived in rural South Australia, 4 had communication disabilities. | A home-based, personalised intervention to learn to use social media; followed by a four month e-mentoring program (included one-to-one and group support provided by mentors through online social media) | Social participation to enhance social networks | CA |
| Raghavendra  [100] | 2018 | Mixed | Social media (Facebook, Flickr, email) | Australia | 9 youth (mean age=17.0 years) with disabilities from two rural Australian communities. | In-person, one-to-one individualised training and support | Social participation | MMAT 16/17 |
| Raghavendra  [101] | 2013 | Mixed | Social media | Australia | 18 youth aged 10–18 years with physical disabilities (resulting from cerebral palsy, physical disability or acquired brain injury) | In-person, one-to-one individualised training and support | Social participation | MMAT 16/17 |
| Raghavendra  [102] | 2015 | Mixed | Social media (eg Instagram, SpokenPhoto, Facebook, incredimail, gmail, i-message, blog, Skype) | Australia | 8 young people (mean age 15.4 years) with communication disabilities participated from two rural Australian towns | In-person, one-to-one individualised training and support | Social participation | MMAT 16/17 |
| Raghavendra  [103] | 2019 | Mixed | Social media | Australia | 5 students with intellectual/developmental disabilities (aged 12 and 13) from a special school | In-person, one-to-one individualised training conducted at school over 1 ½ school terms | Social participation and connection | CA |
| Raghavendra  [104] | 2012 | Qual | Social media | Australia | 15 young people with physical disabilities ranging from 11–18 years of age, with a mean age of 14.6 years | N/A | Social participation | JBI Qual 7/10 |
| Robertson  [105] | 2016 | Mixed | Social media | United States of America | 16 first-year paediatric residents at Saint Louis University (SLU) | One 1-hour interactive lecture and discussion (with a handout provided) | Knowledge of using social media for information-sharing and issues around professionalism | MMAT 8/17 |
| Robinson  [106] | 2018 | Mixed | Social media | Australia | 70 (43 professionals and 27 youth) - two panels consisting of Australian youth advocates; and international suicide prevention researchers and media and communications specialists. The professional panel consisted of a broad panel of international experts including members from Australia, Austria, Canada, Estonia, Hong Kong, Ireland, New Zealand, South Korea, Switzerland, the United Kingdom and the United States. | N/A | Safe communication about suicide online | MMAT 16/17 |
| Rogers  [107] | 2019 | Quant | Social media | United States of America | 97 university students | An automated PowerPoint presentation (STEPS-TECH, 22 minutes and 33 seconds long) | Sleep | PEDro-P 5/11 |
| Schwertel  [108] | 2014 | Quant | Social media (blog, Facebook, Wiki) | Germany | 124 vocational students from IT sector | N/A | Social media skills | CA |
| Stott  [109] | 2016 | Quant | Social media | United States of America | 14 participants who had a role as: a state-level training administrator, or as a director, manager, contracted provider, university partner, or a nominated representative of the state level training administrator | N/A | Professional use of social media | CEBM CAS 5/12 |
| Tsaousides  [110] | 2011 | Mixed | Facebook | United States of America | 96 people with TBI completed the survey (60% female, age range: 23–70). | N/A | Social participation | MMAT 12/17 |
| Usoro  [111] | 2016 | Qual | Social media | United Kingdom | 18 people with learning disabilities and seven trainers & Local Area Coordinators (LACs) | Five interactive ‘Experience Labs’ were conducted over a period of five months. | Online safety | JBI Qual 2/10 |
| Wang  [112] | 2016 | Quant | Social media (email) | United States of America | 10 students with cognitive disability from a university-affiliated lab school (mean age = 19.3; SD = 1.2) and 10 teacher candidates in a university teacher education programme participated in the study | 15 weeks of e-pal programme (student dyads with weekly guidance provided) | Social communication (electronic writing skills) | JBI QE 8/9 |
| Weaver  [113] | 2019 | Qual | Social media | United States of America | 1 adolescent (15 yo female) | Synchronized practice intervention that integrates the three axioms of mindfulness (attention, intention, and attitude) with an evidence-based mindfulness technique (e.g., deep breathing). | Psychological health and well-being (problematic social media use) | JBI Qual 3/10 |
| Williams  [114] | 2013 | Quant | Social media | United States of America, Canada | 38 program directors for all the training programs and institutes approved by the Commission on Accreditation for Marriage and Family Therapy Training (COMAFTE) within the United States and Canada | N/A | Professional use of social media (student professionals) | CEBM CAS 5/12 |

Table Key: AAC = Augmentative and Alternative Communication; ADHD = Attention Deficit Hyperactivity Disorder; CA = Conference abstract with limited information therefore unable to critically appraise methods; CEBM CAS = Center for Evidence-Based Management Critical Appraisal of a Survey checklist; DSO = Disability Support Organization; ICT = Information and communications technology; IT = Information technology; JBI Qual = JBI Checklist for Qualitative Research; JBI QE = JBI Checklist for Quasi-Experimental Studies; JBI C = JBI Checklist for Cohort Studies; Mixed = Mixed methods research design; MMAT = Mixed Methods Appraisal Tool; N/A = not applicable; PEDro-P = PEDro-P Scale for Critical Appraisal of Group Comparison Studies; Qual = Qualitative research design; Quant = Quantitative research design.
