# Supplementary material for: Training Resources Targeting Social Media Skills to Inform Rehabilitation for People Who Have an Acquired Brain Injury: Scoping Review
Source: J Med Internet Res. 2022 Apr 28;24(4):e35595. doi: 10.2196/35595 (PMC9100544; doi:10.2196/35595)
Supplement: Multimedia Appendix 4 [file jmir_v24i4e35595_app4.docx]

|  | **Website name** | **Author/s** | **URL** | **Audience** | **Content Type** | **Content Topic/s** | **Readability (grade level)** | **Siteimprove issues** | **Siteimprove severity** |
| --- | --- | --- | --- | --- | --- | --- | --- | --- | --- |
| 1 | #OPENCPD | University of Birmingham | <http://opencpd.net/course.html> | Educators | Course | Youth Wellbeing | 11 | 24 | 13 errors  3 warnings  8 review items |
| 2 | AAP News & Journals Gateway | David L. Hill | <https://www.aappublications.org/news/2020/01/07/masteringmedia010720> | Educators/ Paediatricians | Text | Youth Wellbeing | 10 | 26 | 13 errors  5 warnings  8 review items |
| 3 | Adobe | Adobe | <https://www.adobe.com/au/creativecloud/social-media.html> | Anyone | Text | How to (content creation) | 7 | 40 | 20 errors  7 warnings  13 review items |
| 4 | Adobe | Brian Wood,  Meg Hunt | <https://helpx.adobe.com/nz/photoshop/how-to/add-text-pictures.html> | Anyone | Text/ Video | How to (content creation - Photoshop) | 7 | 37 | 20 errors  5 warnings  12 review items |
| 5 | Association for the Advancement of Computing in Education (AACE Review) | [Stefanie Panke](https://www.aace.org/review/author/stefanie-panke/) | <https://www.aace.org/review/why-teach-social-media/> | Educators | Text | Youth Wellbeing on Social Media | 9 | 38 | 24 errors  5 warnings  9 review items |
| 6 | Australian Government | Be Connected, Australian Government | <https://beconnected.esafety.gov.au/topic-library> | Anyone | Text / Resource Library | How to (Internet; Social media) | 6 | 19 | 7 errors  3 warnings  9 review items |
| 7 | Brisbane City Council | Brisbane City Council | <https://www.brisbane.qld.gov.au/things-to-see-and-do/council-venues-and-precincts/libraries/library-programs/free-digital-literacy-training> | Anyone | Text / Workshop | How to (Digital literacy) | 12 | 24 | 13 errors  4 warnings  7 review items |
| 8 | Click Voyagers | Xanthi | <https://clickvoyagers.com/2019/03/25/how-to-make-a-twitter-cover-using-canva/> | Anyone | Text | How to (content creation - Twitter) | 7 | 30 | 19 errors  4 warnings  7 review items |
| 9 | Click Voyagers | Xanthi | <https://clickvoyagers.com/2019/03/22/how-to-use-canva-to-make-a-facebook-cover-for-beginners/> | Anyone | Text | How to (content creation - Facebook) | 6 | 32 | 21 errors  4 warnings  7 review items |
| 10 | Click Voyagers | Xanthi | <https://clickvoyagers.com/2019/03/21/how-to-make-great-instagram-stories-that-engage-people/> | Anyone | Text | How to (content creation - Instagram) | 7 | 30 | 19 errors  4 warnings  7 review items |
| 11 | Click Voyagers | Xanthi | <https://clickvoyagers.com/2018/09/20/how-to-use-igtv-tutorial-and-ideas-for-videos/> | Anyone | Text | How to (Instagram) | 7 | 29 | 18 errors  4 warnings  7 review items |
| 12 | Click Voyagers | Xanthi | <https://clickvoyagers.com/2018/10/21/the-absolute-beginner-parent-guide-to-snapchat/> | Parents | Text | How to (Snapchat) | 6 | 30 | 19 errors  4 warnings  7 review items |
| 13 | Click Voyagers | Xanthi | <https://clickvoyagers.com/2018/08/22/how-to-re-post-an-instagram-story-super-fast-tutorial/> | Anyone | Text | How to (Instagram) | 8 | 30 | 19 errors  4 warnings  7 review items |
| 14 | Click Voyagers | Xanthi | https://clickvoyagers.com/category/social-media-tutorials/ | Anyone | Text | How to (Social media) | 7 | 24 | 15 errors  3 warnings  6 review items |
| 15 | Digital Learn | Megan Grady | <https://www.digitallearn.org/courses/intro-to-facebook> | Anyone | Text | How to (Facebook) | 2 | 9 | 1 error  3 warnings  5 review items |
| 16 | Education Technology | James Higgins | <https://edtechnology.co.uk/Article/online-course-helps-teachers-understand-mental-health-impact-of-social-media/> | Teachers | Text/ Course | Youth Wellbeing | 9 | 17 | 6 errors  4 warnings  7 review items |
| 17 | eLearning Learning | Alankrita | <https://www.elearninglearning.com/social-media/?open-article-id=11285786&article-title=effects-of-social-media-on-your-education-and-career-growth&blog-domain=yourtrainingedge.com&blog-title=your-training-edge> | Students/Adults | Text | Wellbeing; How to (Blog Posts) | 7 | 18 | 8 errors  6 warnings  4 review items |
| 18 | eSafety Key Issue: How to use social media and online chat | eSafety Commissioner, Australian Government | <https://www.esafety.gov.au/key-issues/how-to/social-media-online-chat> | Adults | Text | Cybersafety | 9 | 18 | 4 errors  3 warnings  11 review items |
| 1920 | eSafetyeducation | eSafety Commissioner, Australian Government | <https://www.esafety.gov.au/educators> | Educators | Text | Cybersafety | 9 | 18 | 4 errors  3 warnings  11 review items |
| 21 | eSafetykids | eSafety Commissioner, Australian Government | <https://www.esafety.gov.au/kids> | Kids | Text | Cybersafety | 9 | 18 | 4 errors  3 warnings  11 review items |
| 22 | eSafetyparents | eSafety Commissioner, Australian Government | <https://www.esafety.gov.au/parents> | Parents | Text | Cybersafety | 9 | 18 | 4 errors  3 warnings  11 review items |
| 23 | eSafetyseniors | eSafety Commissioner, Australian Government | <https://www.esafety.gov.au/seniors> | Seniors | Text | Cybersafety | 9 | 18 | 4 errors  3 warnings  11 review items |
| 24 | eSafetywomen | eSafety Commissioner, Australian Government | <https://www.esafety.gov.au/women> | Women | Text | Cybersafety | 9 | 18 | 4 errors  3 warnings  11 review items |
| 25 | eSafetyyoungpeople | eSafety Commissioner, Australian Government | <https://www.esafety.gov.au/young-people> | Young people | Text | Cybersafety | 9 | 18 | 4 errors  3 warnings  11 review items |
| 26 | Eventbrite | [Alan Hennessy (Kompass Media](https://www.eventbrite.ie/o/alan-hennessy-kompass-media-14419805983)) | <https://www.eventbrite.ie/e/linkedin-intensive-training-course-tickets-92935898775> | Adults | Course | How To (LinkedIn) | 7 | 48 | 31 errors  6 warnings  11 review items |
| 27 | Go DIGI | Go Digi | <https://www.godigi.org.au/guides> | Anyone | Guides | How to (Social media) | 2 | 21 | 12 errors  3 warnings  6 review items |
| 28 | HOBO UK SEO Services | Shaun Anderson | <https://www.hobo-web.co.uk/how-to-get-started-in-social-media/> | Anyone | Text | How to (Social media) | 7 | 12 | 8 errors  2 warnings  2 review items |
| 29 | Independent Education Today | James Higgins | <https://ie-today.co.uk/Article/university-of-birmingham-project-helps-teachers-understand-effects-of-social-media/> | Educators | Text/  Course | Youth Wellbeing | 9 | 20 | 8 errors  4 warnings  8 review items |
| 30 | Investment News | Scott Kleinberg | <https://www.investmentnews.com/what-you-can-learn-about-social-media-when-you-never-stop-learning-79220> | Adults | Text | How to (Social media) | 7 | 25 | 14 errors  4 warnings  7 review items |
| 31 | Learn My Way | Good Things Foundation | <https://www.learnmyway.com/subjects> | Anyone | Course | How to (Internet) | 8 | 14 | 6 errors  3 warnings  5 review items |
| 32 | Medium | Victor Camon | <https://medium.com/the-ascent/the-best-lesson-you-can-learn-about-social-media-82068758ae00> | Anyone | Text | Wellbeing | 6 | 19 | 9 errors  2 warnings  8 review items |
| 33 | Miklagard | Milklagard SEO Team | <https://miklagard.dk/blog/social-media-basics/> | Anyone | Text | How to (Social media) | 8 | 18 | 7 errors  3 warnings  8 review items |
| 34 | Northern Beaches & Mosman College | Briana Graydon | <https://nbmc.nsw.edu.au/course/How_to_Set_Up_Facebook> | Adults | Course | How to (Facebook) | 7 | 36 | 21 errors  5 warnings  10 review items |
| 35 | Northern Beaches & Mosman College | Briana Graydon | <https://nbmc.nsw.edu.au/course/How_Set_Up_Instagram> | Adults | Course | How to (Instagram) | 7 | 36 | 21 errors  5 warnings  10 review items |
| 36 | Northern Beaches & Mosman College | Briana Graydon | <https://nbmc.nsw.edu.au/course/Understanding_Hash_Tags> | Adults | Course | How to (Hashtags) | 7 | 36 | 21 errors  5 warnings  10 review items |
| 37 | Promo Republic | Irene Wind | <https://promorepublic.com/en/blog/beginners-guide-instagram-stories/> | Anyone | Text | How to (Instagram) | 8 | 22 | 12 errors  3 warnings  7 review items |
| 38 | Psychological Today | Ben Stoviak | <https://www.psychologytoday.com/us/blog/the-gay-agenda/201907/dear-adults-it-s-time-learn-how-use-social-media> | Parents | Text | Cybersafety | 10.5 | 29 | 18 errors  3 warnings  8 review items |
| 39 | Royal College of Nursing | David Atkinson | <https://rcni.com/learning-disability-practice/newsroom/research-news/social-media-risks-people-who-have-learning-disabilities-154876> | Anyone | Text | Cybersafety | 11 | 33 | 22 errors  3 warnings  8 review items |
| 40 | Tech Onroid | Tech Onroid | <https://www.techonroid.com/social-media-tutorials-for-beginners/> | Anyone | Text | How to (Social media) | 7 | 33 | 20 errors  4 warnings  9 review items |
| 41 | Tech Pixies | Laura Fox | <https://techpixies.com/episode26/> | Employees | Text/ Podcast | How to (LinkedIn) | 6 | 17 | 6 errors  5 warnings  6 review items |
| 42 | The eSafety Guide | eSafety Commissioner, Australian Government | <https://www.esafety.gov.au/key-issues/esafety-guide> | Adults | Text | Cybersafety | 9 | 18 | 4 errors  3 warnings  11 review items |
| 43 | ThinkYouKnow.org.au | Australian Federal Police | <https://www.thinkuknow.org.au/resources/guides> | Anyone | Text | Cybersafety | 1 | 14 | 4 errors  2 warnings  8 review items |
| 44 | wp beginner | Editorial Staff | <https://www.wpbeginner.com/wp-tutorials/how-to-add-social-share-buttons-in-wordpress/> | anyone | Text | How to (content creation - WordPress) | 7 | 30 | 17 errors  5 warnings  8 review items |
| 45 | YouTube | Hootsuite | <https://www.youtube.com/watch?v=Bg1BcNDXBi4> | Adults | Video | How to (Hootsuite) | 17 | 49 | 25 errors  3 warnings  21 review items |
| 46 | YouTube | Photoshop Tutorial (PSt) | <https://www.youtube.com/watch?v=fMPROcqmq0E> | Anyone | Video | How to (content creation - Photos) | 19 | 52 | 28 errors  3 warnings  21 review items |
| 47 | YouTube | Meredith Marsh | <https://www.youtube.com/watch?v=qQSCkp9ElXY> | Anyone | Video | How to (content creation - Video Editing) | 20 | 50 | 26 errors  3 warnings  21 review items |
| 48 | YouTube | Ferdy Korpershek | <https://www.youtube.com/watch?v=f3hjGCcPvXo> | Anyone | Video | How to (Website Making) | 18 | 48 | 24 errors  3 warnings  21 review items |
