# Supplementary material for: Training Resources Targeting Social Media Skills to Inform Rehabilitation for People Who Have an Acquired Brain Injury: Scoping Review
Source: J Med Internet Res. 2022 Apr 28;24(4):e35595. doi: 10.2196/35595 (PMC9100544; doi:10.2196/35595)
Supplement: Multimedia Appendix 5 [file jmir_v24i4e35595_app5.docx]

| **Name** | **Reference Link** | **Location** | **Target audience** | **Platform** | **How to join** | **Search Term/s** |
| --- | --- | --- | --- | --- | --- | --- |
| 1 PINK Concussions' Women's Group | <https://www.facebook.com/groups/PINKconcussionsWomen/> | United States | Women over age 25 with brain injury | Facebook Group | Request to Join Group | TBI online support groups |
| 2 PINK Concussions Young Women | <https://www.facebook.com/groups/PINKunder25/> | Doesn't specify | Women under age of 25 with brain injury | Facebook Group | Request to Join Group | TBI online support groups |
| 3 PINK Concussions Caregivers | <https://www.facebook.com/groups/PINKconcussionsParents/> | Doesn't specify | Family and carers of females (adult or child) with a brain injury | Facebook Group | Request to Join Group | TBI online support groups |
| 8 PINK Concussions Canada | <https://www.facebook.com/groups/PINKcanada/> | Canada | Women in Canada with brain injury, concussions or PCS | Facebook Group | Request to Join Group | TBI online support groups |
| ABI Support Group | <https://www.facebook.com/groups/182019855763312/> | United States | People with brain injury, families, carers | Facebook Group | Closed group - go to Facebook group page and click 'Join group' | ABI TBI |
| ABI, TBI survivor's | <https://www.facebook.com/groups/1959491667516184/> | Doesn't specify | People with brain injury | Facebook Group | Closed group - go to Facebook group page and click 'Join group' | ABI TBI |
| ABI/TBI Awareness | <https://www.facebook.com/groups/363999140334180> | Doesn't specify | People with brain injury, families, carers | Facebook Group | Public group - go to Facebook group page and click 'Join group' | ABI TBI |
| ACQUIRED BRAIN INJURY | <https://www.facebook.com/groups/STOCKIE/> | Doesn't specify | People with Brain injury | Facebook Group | Closed group - go to Facebook group page and click 'Join group' | brain injury support |
| Acquired Brain Injury (ABI) Australia | <https://www.facebook.com/groups/683073638862419/> | Australia | People with brain injury | Facebook Group | Closed group - go to Facebook group page and click 'Join group' | brain injury support |
| Acquired Brain Injury Support | <https://www.facebook.com/groups/337608946367602/> | Doesn't specify | People with brain injury, families, carers | Facebook Group | Closed group - go to Facebook group page and click 'Join group' | brain injury support |
| Amy's TBI Tribe \| Concussion & Brain Injury | <https://www.facebook.com/groups/792052120888627/> | Doesn't specify | People with brain injury, families, carers | Facebook Group | Closed group - go to Facebook group page and click 'Join group' | brain injury support |
| Australia and New Zealand TBI Tribe with Leanne Whitehouse | <https://www.facebook.com/groups/ANZTBITribewithLeanneWhitehouse/> | Australia & New Zealand | People with brain injury, families, carers | Facebook Group | Closed group - go to Facebook group page and click 'Join group' | ABI TBI |
| Beautifully Broken (TBI, ABI, Stroke, PTSD and Caregivers support group) | <https://www.facebook.com/groups/579025435853233/> | Doesn't specify | People with brain injury, families, carers | Facebook Group | Closed group - go to Facebook group page and click 'Join group' | ABI TBI |
| BIAIA Persons affected by brain injury community! | <https://www.facebook.com/groups/BIASurvivorAndFamily/> | United States | People with brain injury, families, carers | Facebook Group | Closed group - go to Facebook group page and click 'Join group' | brain injury support |
| BIM (Brain Injury Matters) | <https://www.facebook.com/groups/693992393986515/> | Australia | People with brain injury | Facebook Group | Public group - go to Facebook group page and click 'Join group' | brain injury support |
| BIRU: brain injury peer support and new beginnings | <https://www.facebook.com/groups/2277159285852519/> | Doesn't specify | People with brain injury, families, carers | Facebook Group | Closed group - go to Facebook group page and click 'Join group' | brain injury support |
| BITES (Brain Injury Technology and Education Supports) | <https://www.facebook.com/groups/BITESGroup/> | United States | People with brain injury | Facebook Group | Closed group - go to Facebook group page and click 'Join group' | brain injury support |
| Brain Aneurysm Survivors Australia | <https://www.facebook.com/groups/216646688815844/> | Australia | People with brain injury (resulting from an anuerysm), families, carers | Facebook Group | Closed group - go to Facebook group page and click 'Join group' | brain injury support |
| Brain aneurysm,strokes, AVM survivors and caregivers | <https://www.facebook.com/groups/1669335320015892/> | Doesn't specify | People with brain injury, families, carers | Facebook Group | Closed group - go to Facebook group page and click 'Join group' | brain injury support |
| Brain Balance- Brain Injury Support Group | <https://www.facebook.com/groups/323897314866498/> | United States | People with brain injury | Facebook Group | Closed group - go to Facebook group page and click 'Join group' | brain injury support |
| Brain Injury | <https://www.facebook.com/groups/2441101405> | Doesn't specify | People with brain injury | Facebook Group | Public group - go to Facebook group page and click 'Join group' | brain injury support |
| Brain Injury | <https://www.facebook.com/groups/2365249728/> | Doesn't specify | People with brain injury, families, carers | Facebook Group | Closed group - go to Facebook group page and click 'Join group' | brain injury support |
| Brain Injury & Mental Health Support | https://www.facebook.com/groups/braininjurysupportuk/ | Global | People with brain injury, families, carers | Facebook Group | Closed group - go to Facebook group page and click 'Join group' | brain injury support |
| Brain Injury Alliance of Kentucky™ | <https://www.facebook.com/groups/131994790182/> | United States | People with brain injury, families, carers | Facebook Group | Public group - go to Facebook group page and click 'Join group' | brain injury support |
| Brain Injury and Mental Health - sharing, caring, understanding | <https://www.facebook.com/groups/346547919414221/> | Doesn't specify | People with brain injury | Facebook Group | Closed group - go to Facebook group page and click 'Join group' | brain injury support |
| Brain Injury Association (BIA) | <https://www.facebook.com/groups/2235901986> | Doesn't specify | People with brain injury | Facebook Group | Public group - go to Facebook group page and click 'Join group' | brain injury support |
| Brain Injury Association of America | <https://www.biausa.org/public-affairs/media/virtual-support-groups> | United States | People with brain injury, families, carers | Virtual support groups | Scroll through the list to find virtual support groups |  |
| Brain Injury Association of Indiana | <https://www.facebook.com/groups/366749033267/> | United States | People with brain injury, families, carers | Facebook Group | Closed group - go to Facebook group page and click 'Join group' | brain injury support |
| Brain Injury Awareness | <https://www.facebook.com/groups/BrainInjuryAwarenessSupportGroup/> | Doesn't specify | People with brain injury, families, carers | Facebook Group | Closed group - go to Facebook group page and click 'Join group' | brain injury support |
| Brain Injury Awareness and Support Queensland | <https://www.facebook.com/groups/1724856447771556/> | Australia | People with brain injury, families, carers | Facebook Group | Closed group - go to Facebook group page and click 'Join group' | brain injury support |
| Brain Injury Awareness UK | <https://www.facebook.com/groups/127755034557497/> | United Kingdom | People with brain injury, families, carers | Facebook Group | Closed group - go to Facebook group page and click 'Join group' | brain injury support |
| Brain Injury Family Support Group | <https://www.facebook.com/groups/braininjuryfamilysupport/> | Doesn't specify | Family and carers of people with brain injury | Facebook Group | Closed group - go to Facebook group page and click 'Join group' | brain injury support |
| Brain Injury Help, News, and Support Community | <https://www.facebook.com/groups/brain.injury.help.news.and.support.community/> | Doesn't specify | People with brain injury, families, carers | Facebook Group | Closed group - go to Facebook group page and click 'Join group' | brain injury support |
| Brain Injury Help, News, and Support Community Group | <https://www.facebook.com/groups/brain.injury.help.news.and.support.community/> | United States | People with brain injury, families, carers | Facebook Group | Closed group - go to Facebook group page and click 'Join group' | brain injury online support groups |
| Brain Injury Network | <https://facebook.com/braininjurynetwork> | United States | People with brain injury | Facebook Page | Open page. LIKE the Brain Injury Network official Facebook page at:  https://facebook.com/braininjurynetwork | brain injury online support groups |
| Brain Injury Network Facebook Support Group | <https://www.facebook.com/sabisue> | United States | People with brain injury | Facebook Group | Closed group - go to Facebook group page and click 'Join group'. | brain injury online support groups |
| Brain Injury Peer Support Closed Facebook Group for Families/Carers | <https://www.facebook.com/pg/braininjurytasmania/groups/?ref=page_internal> | Australia | Families and Carers of people with brain injury | Facebook Group | Closed group. If you would like to join one of these groups please visit the BIAT Facebook Page | brain injury online support groups |
| Brain Injury Peer Support Closed Facebook Group for Individuals | <https://www.facebook.com/pg/braininjurytasmania/groups/?ref=page_internal> | Australia | People with brain injury | Facebook Group | Closed group. If you would like to join one of these groups please visit the BIAT Facebook Page | brain injury online support groups |
| Brain Injury SUCKS! | <https://www.facebook.com/groups/707975209230063/> | Doesn't specify | People with brain injury | Facebook Group | Closed group - go to Facebook group page and click 'Join group' | brain injury support |
| Brain Injury Support - Teamhilevel | <https://www.facebook.com/groups/teamhilevel/> | United States | People with brain injury, families, carers | Facebook Group | Closed group - go to Facebook group page and click 'Join group' | brain injury support |
| Brain Injury Support Group | <https://www.dailystrength.org/group/brain-injury> | Doesn't specify | People with brain injury, families, carers | Online Forum | Add New Post to online page | "brain injury online support groups" |
| Brain Injury Support Group | <https://www.facebook.com/pages/category/Community/Brain-Injury-Support-Group-163713670318087/> | Doesn't specify | People with brain injury, families, carers | Facebook Group | Open group- anyone can like, post, or join | brain injury online support groups facebook |
| Brain Injury Support Group | <https://www.facebook.com/groups/braininjurysupportgroup/> | Doesn't specify | People with brain injury, families, carers | Facebook Group | Closed group - go to Facebook group page and click 'Join group' | brain injury support |
| Brain Injury Support Group for Survivors and their Families | <https://www.facebook.com/groups/209849259174632/> | Doesn't specify | People with brain injury, families, carers | Facebook Group | Closed group - go to Facebook group page and click 'Join group' | brain injury support |
| Brain Injury Support Group of Duluth | <https://www.facebook.com/groups/292271587914620/> | Doesn't specify | People with brain injury | Facebook Group | Closed group - go to Facebook group page and click 'Join group' | brain injury support |
| Brain Injury Support Group-Brainworks 501(c)3 - ASBISG.org | <https://www.facebook.com/groups/BrainInjurySupport/> | United States | People with brain injury, families, carers | Facebook Group | Public group - go to Facebook group page and click 'Join group' | brain injury support |
| Brain Injury Support TBI | <https://www.facebook.com/groups/inspiringdancewalker/> | United States | People with brain injury | Facebook Group | Closed group - go to Facebook group page and click 'Join group' | brain injury support |
| Brain Injury Survivors in Canada | <https://www.facebook.com/groups/1781149405523707/> | Canada | People with brain injury, families, carers | Facebook Group | Public group - go to Facebook group page and click 'Join group' | brain injury support |
| Brain Injury Survivors Support Group | <https://www.facebook.com/groups/473292253355459/> | Doesn't specify | People with brain injury | Facebook Group | Closed group - go to Facebook group page and click 'Join group' | brain injury support |
| Brain Injury Survivors Worldwide Community | <https://facebook.com/brain.injury.survivors.worldwide.community> | United States | People with brain injury | Facebook Page | Open page. LIKE the Brain Injury Survivors Worldwide Community page on Facebook at:   https://facebook.com/brain.injury.survivors.worldwide.community | brain injury online support groups |
| Brain Injury TBI/ABI Warriors and Caregivers | <https://www.facebook.com/groups/458471814795769/> | Doesn't specify | People with brain injury, families, carers | Facebook Group | Closed group - go to Facebook group page and click 'Join group' | ABI TBI |
| Brain Injury The New Me? Australia | <https://www.facebook.com/groups/braininjurythenewmeaustralia/> | Australia | People with brain injury | Facebook Group | Closed group - go to Facebook group page and click 'Join group' | brain injury support |
| BRAIN INJURY UNFILTERED / TBI & PCS Survivors | <https://www.facebook.com/groups/1909226822427951/> | Doesn't specify | People with brain injury | Facebook Group | Closed group - go to Facebook group page and click 'Join group' | brain injury support |
| Brain Injury warriors n survivors support group India. The Invisible Injury | <https://www.facebook.com/groups/1556895054562130/> | India | People with brain injury, families, carers | Facebook Group | Public group - go to Facebook group page and click 'Join group' | brain injury support |
| Brain Injury Warrior's Support and Safe Place | <https://www.facebook.com/groups/3094705360584849/> | Doesn't specify | People with brain injury, families, carers | Facebook Group | Closed group - go to Facebook group page and click 'Join group' | brain injury support |
| Brain Tumour Support Group Australia | <https://www.facebook.com/groups/198016110233965/> | Australia | People with brain injury (resulting from brain cancer of tumour) | Facebook Group | Closed group - go to Facebook group page and click 'Join group' | brain injury support |
| Brainstorm For Brain Injury Support Group | <https://www.facebook.com/groups/423867728219969/> | Doesn't specify | People with brain injury, families, carers | Facebook Group | Public group - go to Facebook group page and click 'Join group' | brain injury support |
| Brainworks 501(c)3 ASBISG.org Brain Injury/Neurological Support Org | <https://www.facebook.com/groups/ASBISGSupportGroup/> | United States | People with brain injury, families, carers | Facebook Group | Closed group - go to Facebook group page and click 'Join group' | brain injury support |
| Building Your Life After Traumatic Brain Injury | <https://www.facebook.com/groups/buildingyourlifeaftertraumaticbraininjury/> | United States | People with brain injury, families, carers | Facebook Group | Closed group - go to Facebook group page and click 'Join group' | brain injury support |
| 'Caregivers & Parents' Group - Post Concussion Syndrome & MTBI Awareness | <https://www.facebook.com/groups/caregiversandparentsPCSAWW> | Doesn't specify | Family and carers of people with brain injury (post concussion syndrome) | Facebook Group | Closed group - go to Facebook group page and click 'Join group' | brain injury support |
| Caregivers and Family of ABI/TBI Survivors | <https://www.facebook.com/groups/BISfam/> | Doesn't specify | Family and carers of people with brain injury | Facebook Group | Closed group - go to Facebook group page and click 'Join group' | ABI TBI |
| Carer's of People with an Acquired Brain Injury - Support Group | <https://www.facebook.com/groups/416379471806758/> | Doesn't specify | Family and carers of people with brain injury | Facebook Group | Closed group - go to Facebook group page and click 'Join group' | brain injury support |
| Central Queensland ABI TBI survivors & supporters Australia | <https://www.facebook.com/groups/1144424842388425/> | Australia | People with brain injury, families, carers | Facebook Group | Closed group - go to Facebook group page and click 'Join group' | ABI TBI |
| Chatroom | <http://www.tbihome.org/> | Doesn't specify | People with brain injury, families, carers | Online discussion boards | Post on message board or chatroom | traumatic brain injury forum |
| Colorado Brain Injury Support | <https://biacolorado.org/support-groups/> | United States | People with brain injury, families, carers | Online support space | Sign up with Email- contact with further instructions | brain injury online support |
| Community Forums | <https://www.traumasurvivorsnetwork.org/forums> | Doesn't specify | People with brain injury, families, carers | Online forum with discussion posts | Register an account | traumatic brain injury forum |
| Concussion, Post Concussion Syndrome and Brain Injuries Support Group | <https://www.facebook.com/groups/1078165138911832/> | Doesn't specify | People with brain injury | Facebook Group | Closed group - go to Facebook group page and click 'Join group' | brain injury support |
| CTE & Brain Injury Global Support | <https://www.facebook.com/groups/164998687455984/> | Global | People with brain injury, families, carers | Facebook Group | Closed group - go to Facebook group page and click 'Join group' | brain injury support |
| Devon ABI & TBI Parents & Carers Page | <https://www.facebook.com/groups/179693162461740/> | United Kingdom (Devon) | Family and carers of children with brain injury | Facebook Group | Closed group - go to Facebook group page and click 'Join group' | ABI TBI |
| Family Caregiver Alliance | <https://www.caregiver.org/support-groups> | United States | Family and carers of people with brain injury | Email based discussion space | You can subscribe and unsubscribe here: lists.caregiver.org/mailman/listinfo/caregiver-online_lists.caregiver.org | "brain injury support" |
| Family START Group | <https://www.biaoregon.org/services/support-groups/> | United States | Parents of children with brain injury | meeting space for discussion | Fill out Google Form on website | "brain injury online support groups" |
| Genyus | <https://genyusnetwork.com/> | Global | People who have experienced trauma | Story sharing forum | Sign up with email |  |
| Head Injury Caregivers Support Group (PCS, PTSD, ABI, TBI) | <https://www.facebook.com/groups/187440888316911> | Australia | Family and carers of people with brain injury | Facebook Group | Closed group - go to Facebook group page and click 'Join group' | brain injury support |
| Hope After Head Injury Support Group | <https://www.facebook.com/groups/hopeafterheadinjury/> | Doesn't specify | People with brain injury | Facebook Group | Closed group - go to Facebook group page and click 'Join group' | brain injury support |
| Humor Heals! (Brain Injury Support Group) | <https://www.facebook.com/groups/TBIfamily/> | United States | People with brain injury, families, carers | Facebook Group | Closed group - go to Facebook group page and click 'Join group' | brain injury support |
| Injuries and Trauma Forum | <https://www.patientslikeme.com/patients/searches/detail_search> | Doesn't specify | People with brain injury, families, carers | Online forum | Set up an account | brain injury forum |
| LIFE AFTER BRAIN INJURY | <https://www.facebook.com/groups/lifeafterbraininjury/> | Doesn't specify | People with brain injury, families, carers | Facebook Group | Closed group - go to Facebook group page and click 'Join group' | brain injury support |
| Love Your Brain Retreat | <https://www.loveyourbrain.com/retreat> | United States & Canada | People with brain injury, families, carers | Retreat | Sign up Online (Application) | "brain injury support" |
| Mindslide: TBI & PTSD Support Group | <https://www.facebook.com/groups/213887756351665/> | United States | People with brain injury, families, carers | Facebook Group | Public group - go to Facebook group page and click 'Join group' | ABI TBI |
| Mothers With Children Who Have ABI/TBI | <https://www.facebook.com/groups/443458255854682/> | Doesn't specify | Family and carers of children with brain injury | Facebook Group | Closed group - go to Facebook group page and click 'Join group' | ABI TBI |
| Online Brain Injury Peer Support Program meetings | <https://www.biat.org.au/information-referral/brain-injury-peer-support-program> | Australia | People with brain injury, families, carers | Zoom meetings | Sign up with Email- contact with further instructions | brain injury online support groups |
| Online Community | <https://www.aftertrauma.org/forum/index.php> | Doesn't specify | People with brain injury | Online forum with discussion posts | Create an account then Add a discussion post/thread | tbi online forum |
| Online Support for Brain Injury, TBI & ABI | <https://www.facebook.com/groups/Brainscramble/> | Doesn't specify | People with brain injury, families, carers | Facebook Group | Closed group - go to Facebook group page and click 'Join group' | ABI TBI |
| Parents of Children with Brain Injuries | <https://www.facebook.com/groups/ParentsofChildrenwithBrainInjuries/> | Doesn't specify | Parents of children with brain injury | Facebook Group | Closed group - go to Facebook group page and click 'Join group' | brain injury support |
| Parents w/ TBI ABI PCS CTE AVM SAH Stroke Epilepsy Anoxic Tumor BrainInjury | <https://www.facebook.com/groups/1130360083696626/> | United States | People with brain injury (who are also parents) | Facebook Group | Closed group - go to Facebook group page and click 'Join group' | ABI TBI |
| Parkview Brain Injury Support Group | <https://www.facebook.com/groups/975959132563247/> | Doesn't specify | People with brain injury (Private Group - this group is for the participants of Parkview group only) | Facebook Group | Closed group - go to Facebook group page and click 'Join group' | brain injury support |
| Positive thoughts after Stroke,TBI, ABI, Aneurysm and other Brain Injuries | <https://www.facebook.com/groups/PositiveThoughtsGroup/> | Doesn't specify | People with brain injury | Facebook Group | Closed group - go to Facebook group page and click 'Join group' | ABI TBI |
| Post Concussion Syndrome Support Group | <https://www.facebook.com/groups/108398302515255/> | Doesn't specify | People with brain injury (post concussion syndrome) | Facebook Group | Closed group - go to Facebook group page and click 'Join group' | brain injury support |
| Post Traumatic Stress Disorder and Traumatic Brain Injury | <https://www.facebook.com/groups/PTSD.TBI/> | Doesn't specify | People with brain injury | Facebook Group | Closed group - go to Facebook group page and click 'Join group' | brain injury support |
| Saddleback Church Brain Injury Support Group | <https://www.facebook.com/groups/421854437996263/> | United States | People with brain injury, families, carers | Facebook Group | Public group - go to Facebook group page and click 'Join group' | brain injury support |
| Safe Haven: A TBI Group | <https://www.facebook.com/groups/safehaventbi/> | Doesn't specify | People with brain injury, families, carers | Facebook Group | Closed group - go to Facebook group page and click 'Join group' | ABI TBI |
| Smart Patients Brain Injury Support Group | <https://www.smartpatients.com/forums/brain-injury> | Doesn't specify | People with brain injury, families, carers | Online discussion forum | Sign up with email address | brain injury forum |
| Social TBI Community | <https://www.trymunity.com/?gclid=CNG5s5Wx3tQCFYWFfgodj9AOOw> | United States | People with brain injury, families, carers | online support network | Sign up online, make an account | "brain injury online support groups" |
| Supporting A.B.I. (Acquired Brain Injury) | <https://www.facebook.com/groups/2549346291/> | Canada | People with brain injury, families, carers | Facebook Group | Closed group - go to Facebook group page and click 'Join group' | brain injury support |
| Supporting Caregivers Online Group! | <https://www.biav.net/support-groups/> | United States | Family and carers of people with brain injury | Online support space | Sign up on website to make an account | brain injury online support |
| Synapse Forums | <https://synapse.org.au/community-hub/brain-injury-forum/> | Australia | People with brain injury, families, carers | Forum discussion space On Synapse website | Need to set up an account on website |  |
| TBI and ABI (Brain Injury) Depression Guidance and Sanctuary | <https://www.facebook.com/groups/1593947340923726/> | Doesn't specify | People with brain injury, families, carers | Facebook Group | Closed group - go to Facebook group page and click 'Join group' | brain injury support |
| TBI AWARENESS | <https://www.facebook.com/groups/977573282314641> | Doesn't specify | People with brain injury, families, carers | Facebook Group | Public group - go to Facebook group page and click 'Join group' | ABI TBI |
| TBI place | <https://www.facebook.com/groups/331331804155366/> | Doesn't specify | People with brain injury, families, carers | Facebook Group | Public group - go to Facebook group page and click 'Join group' | ABI TBI |
| TBI Support and Awareness | <https://www.facebook.com/TbiAwareness/> | Doesn't specify | People with brain injury, families, carers | Facebook Group | Open group- anyone can like, post, or join | brain injury online support groups facebook |
| TBI Survivors and Caregivers Support Group | <https://www.facebook.com/groups/343140855818138/> | United States | People with brain injury, families, carers | Facebook Group | Closed group - go to Facebook group page and click 'Join group' | brain injury support |
| TBI Survivors and Family/Caregivers Support group | <https://www.facebook.com/groups/1802712906655836/> | United States | People with brain injury, families, carers | Facebook Group | Public group - go to Facebook group page and click 'Join group' | brain injury support |
| TBI survivors successes | <https://www.facebook.com/groups/TBISS/> | Doesn't specify | People with brain injury | Facebook Group | Closed group - go to Facebook group page and click 'Join group' | brain injury support |
| TBI Worldwide - STOP the Silence for Traumatic Brain Injuries | <https://www.facebook.com/groups/315000432186818/> | Doesn't specify | People with brain injury, families, carers | Facebook Group | Public group - go to Facebook group page and click 'Join group' | brain injury support |
| TBI/ABI Caregiver Support Group | <https://www.facebook.com/groups/1245236052220171/> | Doesn't specify | Family and carers of people with brain injury | Facebook Group | Closed group - go to Facebook group page and click 'Join group' | ABI TBI |
| TBI/ABI Friends | <https://www.facebook.com/groups/109418939076893/> | Doesn't specify | People with brain injury, families, carers | Facebook Group | Closed group - go to Facebook group page and click 'Join group' | ABI TBI |
| TBI/ABI/HEAD INJURY "SURVIVOR'S ONLY" GROUP FOR VENTING/TALKING!!! | <https://www.facebook.com/groups/522074011215549/> | United States | People with brain injury | Facebook Group | Closed group - go to Facebook group page and click 'Join group' | ABI TBI |
| The Ghost In My Brain - Traumatic Brain Injury (TBI) Support Group | <https://www.facebook.com/groups/tbisupportgroup/> | United States | People with brain injury, families, carers | Facebook Group | Closed group - go to Facebook group page and click 'Join group' | brain injury support |
| The Severe Brain Injury Caregiver Support Group | <https://www.biamd.org/online-support-groups.html> | United States | Family and carers of people with brain injury | Online discussion space at scheduled times | Contact for information | brain injury online support |
| Together after Brain Injury: Online Support Group | <https://www.facebook.com/groups/BIsupportNC/> | United States | People with brain injury, families, carers | Facebook Group | Closed group - go to Facebook group page and click 'Join group' | brain injury support |
| Traumatic Brain Injuries | <http://www.braininjurysupport.org/forums/> | United States | People with brain injury, families, carers | Online forum with discussion posts | Create an account then Add a discussion post/thread | tbi online forum |
| Traumatic Brain Injury ~~~ T.B.I~~~ TERRIFIC BEYOND INJURY!!!! | <https://www.facebook.com/groups/705621112859581/> | United States | People with brain injury | Facebook Group | Closed group - go to Facebook group page and click 'Join group' | brain injury support |
| Traumatic Brain Injury Awareness - New Zealand/Aotearoa | <https://www.facebook.com/groups/2020005784887287/> | New Zealand/Aotearoa | People with brain injury | Facebook Group | Closed group - go to Facebook group page and click 'Join group' | brain injury support |
| Traumatic Brain Injury Awareness and Support | <https://www.facebook.com/groups/traumaticbraininjuryawareness/> | United States | People with brain injury, families, carers | Facebook Group | Closed group - go to Facebook group page and click 'Join group' | brain injury support |
| Traumatic Brain Injury Healing & Recovery Support Group | <https://www.facebook.com/groups/186712754690242/> | Doesn't specify | People with brain injury, families, carers | Facebook Group | Closed group - go to Facebook group page and click 'Join group' | brain injury support |
| Traumatic Brain Injury Healthy Alternatives | <https://www.facebook.com/groups/418909271603041/> | Doesn't specify | People with brain injury | Facebook Group | Closed group - go to Facebook group page and click 'Join group' | brain injury support |
| Traumatic Brain Injury Support Group | <https://www.facebook.com/Traumatic-Brain-Injury-Support-Group-180684948672011/> | Doesn't specify | People with brain injury, families, carers | Facebook Group | Open group, just like or follow the page and post discussion posts | "brain injury online support groups" |
| Traumatic Brain Injury Support Group | <https://www.facebook.com/groups/122907408341663/> | United States | People with brain injury, families, carers | Facebook Group | Closed group - go to Facebook group page and click 'Join group' | brain injury support |
| Traumatic or Acquired Brain Injury Support Group | <https://www.facebook.com/groups/6367734334/> | Doesn't specify | People with brain injury, families, carers | Facebook Group | Closed group - go to Facebook group page and click 'Join group' | brain injury support |
| Treasure Valley Brain Injury Support Group | <https://www.facebook.com/groups/140517879314097/> | United States | People with brain injury, families, carers | Facebook Group | Closed group - go to Facebook group page and click 'Join group' | brain injury support |
